# Clinicopathological evaluation and survival of patients with squamous cell carcinoma of the tongue

**DOI:** 10.4317/medoral.22421

**Published:** 2018-09-28

**Authors:** Thamirys-Dantas Nóbrega, Salomão-Israel-Monteiro-Lourenço Queiroz, Edilmar-de Moura Santos, Antônio-de Lisboa-Lopes Costa, Leão Pereira-Pinto, Lélia-Batista de Souza

**Affiliations:** 1 Bolsista PIBIC-CNPq/Liga Norte Riograndense Contra o Câncer, Natal/RN Brasil; 2 Doutorando do programa de Pós-Graduação em Patologia Oral da Universidade Federal do Rio Grande do Norte, Natal/RN, Brasil; 3 Membro da Liga Norte Riograndense Contra o Câncer, Natal/RN Brasil; 4 Professor(a) do programa de Pós-Graduação em Patologia Oral da Universidade Federal do Rio Grande do Norte, Natal/RN, Brasil

## Abstract

**Background:**

Early detection of oral cancer is the most effective means of reducing morbidity, complexity, and extent of treatment. This study evaluated the clinicopathological profile of epidermoid carcinoma of the tongue, including treatment and survival.

**Material and Methods:**

This observational, retrospective cross-sectional study evaluated patients with squamous cell carcinoma of the tongue treated at the Dr. Luiz Antônio Hospital, Natal, Brazil, from January 2001 to December 2011. Survival variables were calculated using the Kaplan-Meier method and compared by log rank tests.

**Results:**

Of the 412 patients diagnosed in this period, 298 (72.3%) were men; their mean age was 60.5 years, and 69.2% were diagnosed with stage III/IV tumours. Improved survival was associated with early stage diagnosis, absence of affected lymph nodes at diagnosis, and treatment with surgery alone.

**Conclusions:**

Late stage diagnosis of oral cancer negatively affects patient survival. In addition, the general public should be made aware of the prognostic factors for oral SCC of the tongue and of the importance of periodic examinations of the oral cavity.

** Key words:**Mouth neoplasms, tongue neoplasms, carcinoma, squamous cell, survival analysis.

## Introduction

Cancer is a major cause of morbidity and mortality worldwide, with incidence rates varying widely depending on geographic location, age, sex and race (1). Low and middle income countries have proportionately higher cancer burdens than high income countries, but their health systems are unprepared to address this problem (2).

Oral squamous cell carcinoma (SCC) is the most common type of cancer affecting the oral mucosal epithelium (3). Its worldwide incidence is quite variable, being the main type of cancer in Southeast Asian countries. Cancer of the oral cavity is classified as a type of head and neck tumour. For Brazil, an estimated 11,200 new cases of oral cavity cancer in men and 3,500 in women are estimated for each year of the 2018-2019 biennium. These figures correspond to an estimated risk of 10.86 new cases per 100,000 men, ranking fifth; and 3.28 for every 100,000 women, being the 12th most frequent among all cancers (4).

The main risk factors for the development of SCC are the consumption of tabaco and alcohol and HPV infections, but only a fraction of these exposed people develop this neoplasia (5). These findings suggest that theory that genetic factors are individuals that contribute to greater susceptibility, differences in clinical behaviour and patient prognosis (5,6).

Although oral SCC can develop in various areas of the oral cavity, its highest incidence is in the tongue. Post-surgical recurrence rates are high, as is the propensity to develop regional lymph node metastases. Among patients with oral tumours, those with SCC of the tongue have the poorest survival rates (7,8).

Tumour aggressiveness has been associated with several factors, including the histological grade of malignancy, lesion size, the presence of metastases present at diagnosis and the anatomical location of the tumour (9). Treatment of these tumours, which can include surgery, radiotherapy and/or chemotherapy, depends largely on tumour location, clinical stage, histopathologic grading and the physical condition of the patient. The 5-year survival rate is about 50%, with no major changes in the past three decades, despite advances in treatment (10).

Efforts are needed to evaluate the clinical and pathological parameters and diagnostic methods that can expedite the early detection of oral cancer and thus improve patient prognosis. This study therefore evaluated the clinicopathological characteristics of patients diagnosed with SCC of the tongue and treated at a single centre over an 11-year period, as well as evaluating the survival of these patients.

## Material and Methods

This retrospective, sectional study included patients histopathologically diagnosed with SCC of the tongue who were treated at Dr. Luiz Antonio Hospital, Natal, Brazil, between January 2001 and December 2011. Patients were excluded if they had oral SCC at other sites or if their medical records did not report metastasis, recurrence and survival or if they only underwent incisional biopsy. Data collected from patients’ medical records included sex; age; presence of lymph node metastasis, distant metastasis, or local recurrence; treatment; overall survival; and last record date or date of death. The study protocol was approved by the Research Ethics Committee of the Northern League Human Riograndense Against Cancer (CEP / LIGA (Approval No. 923192/2014), which waived the requirement for informed consent due to the retrospective nature of this study.

All data were analysed statistically using Stata / IC version 12.0 software (Stata Corp, College Station, TX). Survival variables were calculated using the Kaplan-Meier method and compared by log rank tests. Multivariate analysis of factors associated with survival was performed using the Cox stepwise regression model (hierarchical), from the highest to the lowest hazard ratio (HR). Variables selected to enter the model were those having a *p*-value ≤ 0.250 on log rank tests, a *p*-value > 0.05 on proportionalities tests, with risk proportionalities graphs not touching. Models with a *p*-value ≤ 0.05 were considered statistically significant. Analysis of model waste by Sehoenfeld, Cox-Snell and residual score (DFBETA) tests showed that no individual patient result could interfere. Significant associations between clinicopathologic features and outcomes were evaluated using chi-square tests, with a *p*-value ≤ 0.05 considered statistically significant.

## Results

Of the 412 patients included in the study, 298 (72.3%) were men and 114 (27.7%) were women. Their mean age was 60.5 ± 14.1 years (range, 19–100 years). Evaluation of educational level showed that 117 (28.4%) patients were illiterate and 189 (45.9%) had some schooling (primary, secondary or higher); information on the remaining 106 (25.7%) patients was not found in their medical records ( Table 1,  Table 1 continue).

**Table 1:**
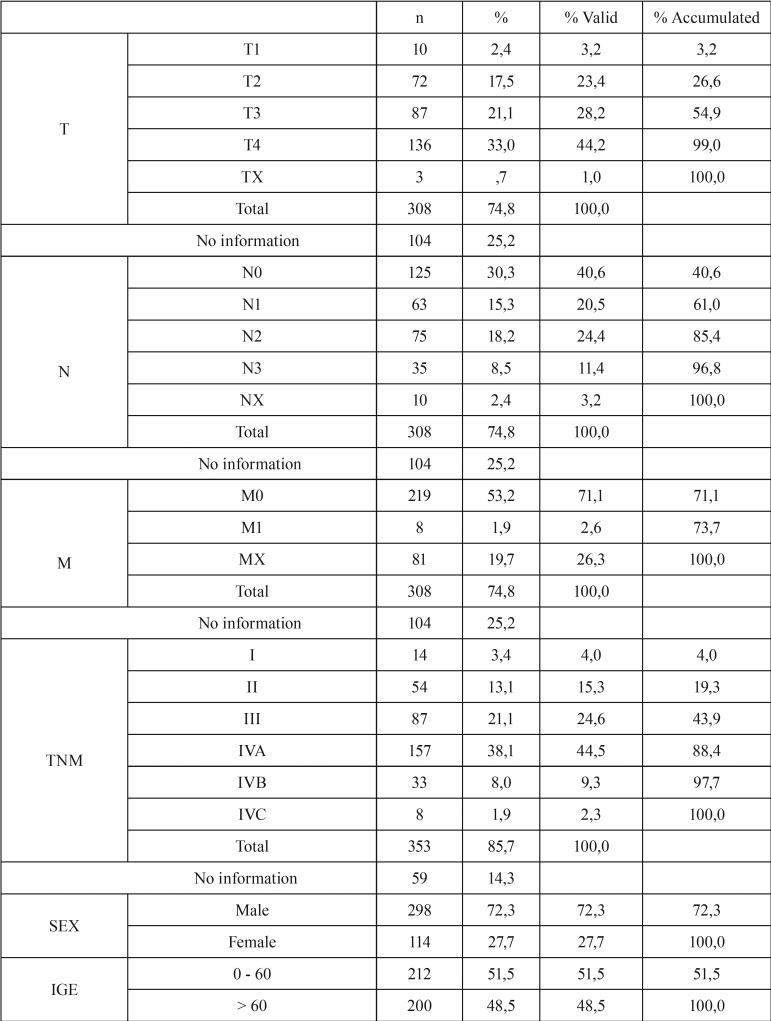
Clinical and demographic data.

**Table 1 continue:**
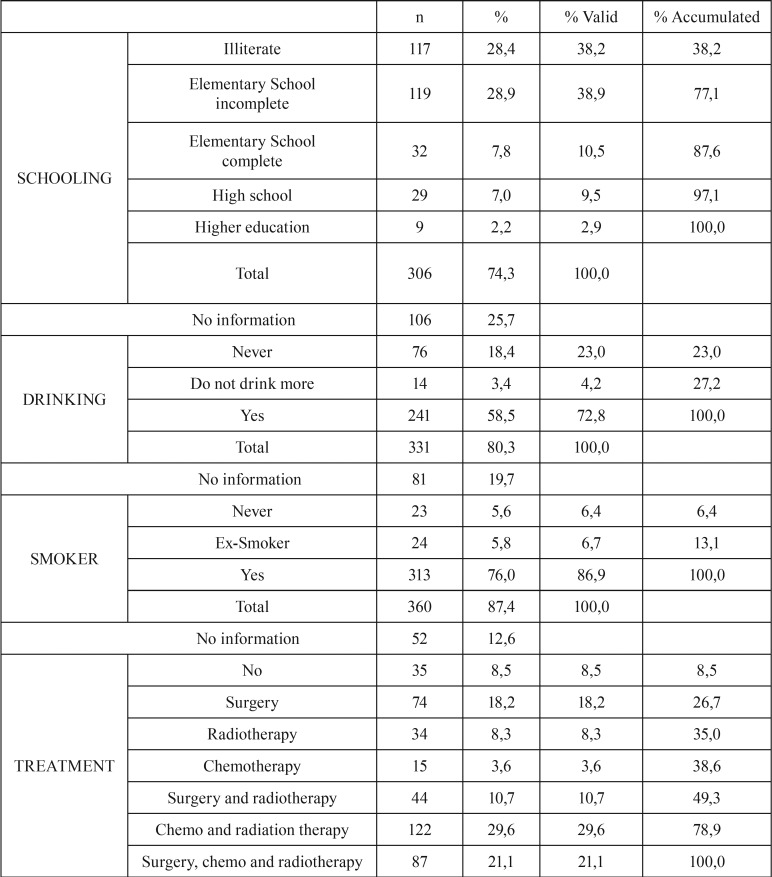
Clinical and demographic data.

Seventeen (4.12%) patients had more than one primary tumour, confirming that these tumours may be present at more than one anatomical site. In addition, 76 (18.45%) patients were diagnosed early, contributing to best survival.

Tobacco use and alcohol consumption were reported by 313 (76%) and 241 (58.5%) patients, respectively. Only 23 (5.6%) patients reported that they were never smokers and 76 (18.4%) stated that they did not consume alcohol. The male sex most associated with smoking (*p* = 0.005, 1.79 times more than women) and alcohol (*p* <0.0001, 4.80 times more than women).

Analysis by type of treatment showed that 35 (8.5%) patients did not undergo any kind of treatment (denial of the patient and terminal stages), 122 (29.6%) underwent chemotherapy and radiation therapy, and 131 (31.8%) underwent surgery and associated treatment. Of 123 patients, 74 (18.2%) underwent surgery alone, 34 (8.3%) received radiotherapy alone and 15 (3.6%) received chemotherapy alone.

Evaluation of TNM classification showed that 223 (54.1%) patients presented with local extension, including 87 (21.1%) classified as T3 and 136 (33%), as T4. Only 10 (2.4%) patients were classified as T1, indicating that few patients are diagnosed at an early stage of the disease. Information on T stage was not available for 59 (14.3%) patients. Assessment of regional spread (N), showed that 125 (30.3%) patients were classified as N0 and 110 (26.7%) as N2 and N3, with information not available for 104 (25.2%) patients. Evaluation of TNM classification showed that 68 (16.5%) were stage I or II and 285 (69.2%) were stage III or IV.

**Figure 1:**
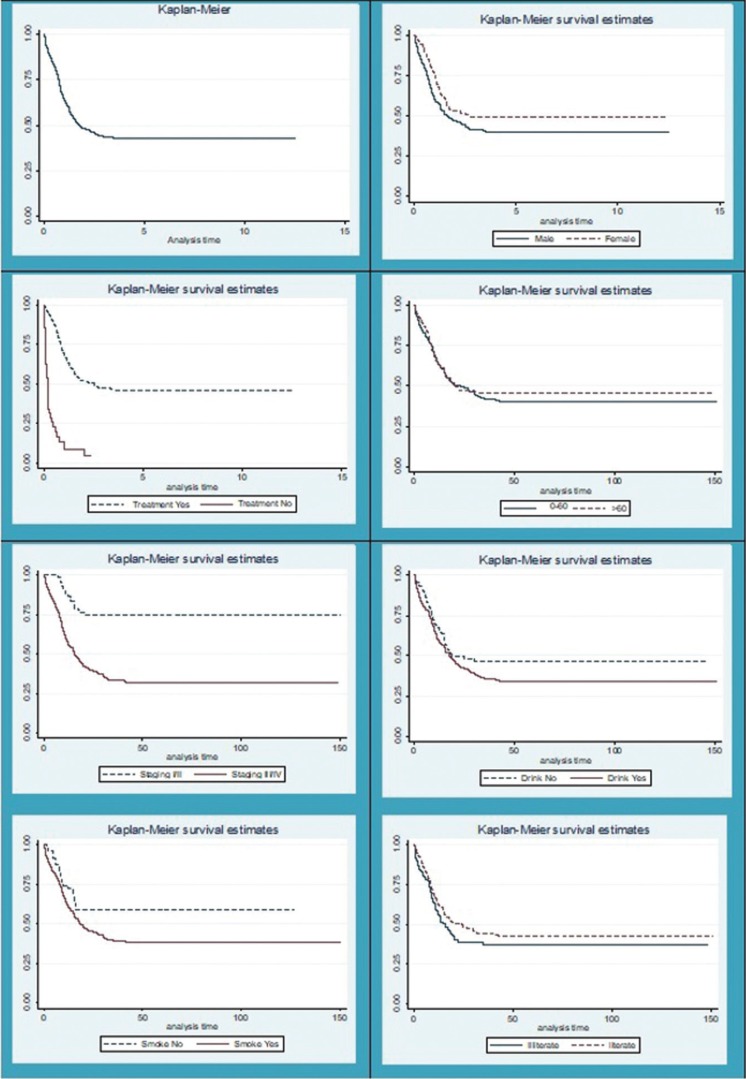
Survival function Kaplan-Meier.

Of the 412 patients, 86 (20.8%) were censored and 224 (54.36%) died within five years. Median overall survival (OS) was 1.77 years (95% confidence interval [CI] 1.42–2.53 years). The 5-year OS rate was 42.61% (95% CI 37.56–47.56%) and remained stable thereafter (Fig. 1).

The mean time of patients who died was 24.6 months. Twenty (8.92%) patients were monitored for ≤ 0.1 month, with the remainder followed-up for 0.1–96 months.

Univariate analysis showed that factors associated with OS included T and N stage (TNM) and treatment (yes/no) (T Table 2). Multivariate analysis using a Cox regression model showed that for only T stage (TNM) and treatment (yes/no) were independent predictors of OS. Poorest survival outcomes were observed in patients with T3 and T4 tumours who did not undergo treatment ( Table 3).

**Table 2:**
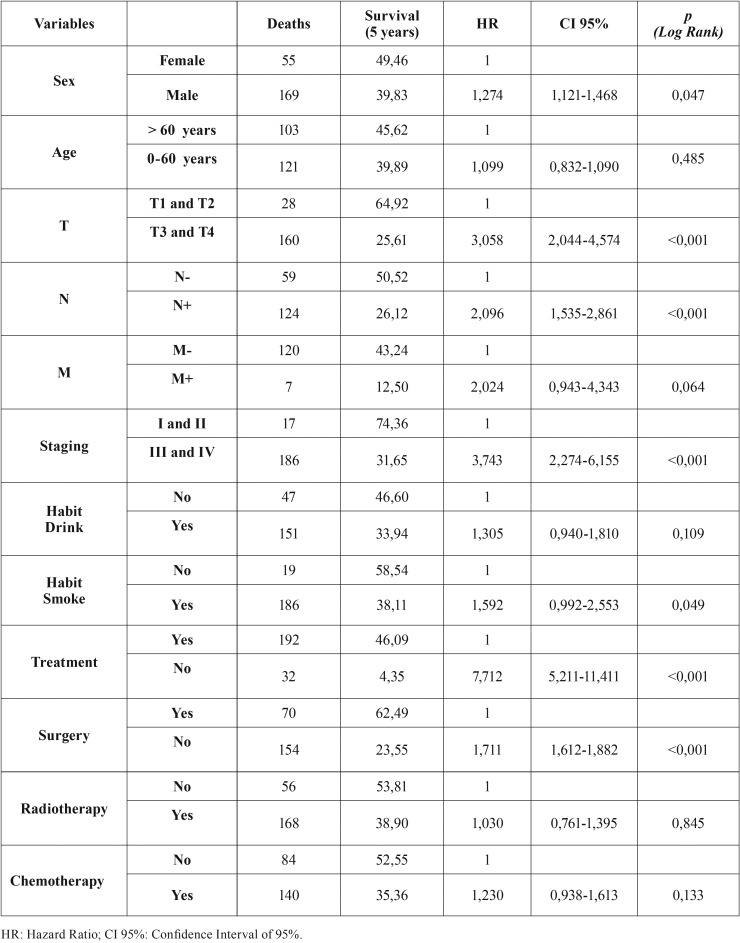
Univariate and proportionality assessment.

**Table 3:**
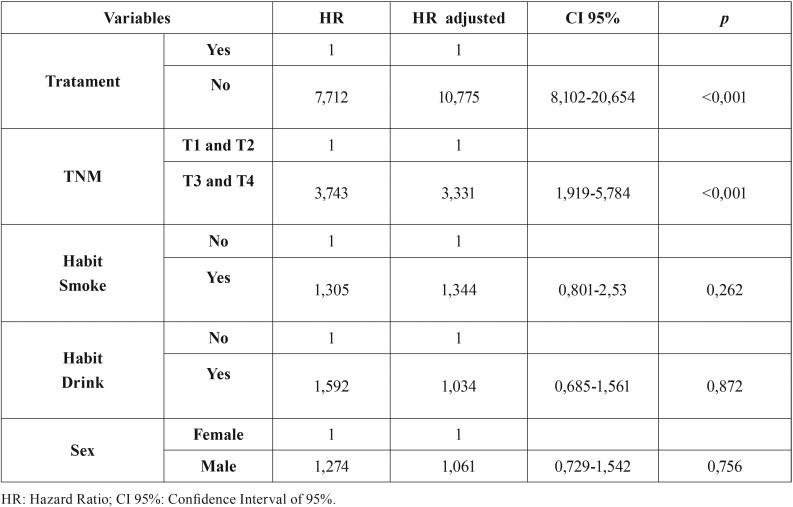
Multivariate analysis with adjusted Cox regression.

Evaluation of the association between clinicopathological features and patient outcomes showed that smoking, treatment (yes/no), surgical treatment, chemotherapy and staging were significantly associated with survival (*p* <0.05 each).

## Discussion

The incidence and mortality rates from oral cancer are high in various populations. Moreover, oral cancer can have a negative effect on patient quality of life, causing problems in swallowing and speaking, as well as significant facial disfigurements that can lead to serious problems in patients’ social lives (2).

SCC of the tongue is the most common cancer of the oral cavity, accounting for 25% to 40% of affected patients. The tongue has a rich lymphatic network and muscle structure, making the tongue the site most often associated with cervical metastases from tumours at other sites in the mouth, resulting in poor patient prognosis (12).

The main risk factors associated with oral SCC of the tongue are genetic predisposition, immune system suppression, nutritional deficiencies, and exposure to the carcinogenic effects of smoking and alcohol consumption (6). These factors can have a synergistic effect, with the incidence of oral SCC of the tongue being directly proportional to the amount and duration of exposure. Other factors as solar radiation exposure and viruses infections, including human papilloma virus (HPV) which are more specific of other SCC, respectively the lip and oropharynx (1,5,11). The majority of studies of oral SCC found that all reported alcohol consumption and/or smoking tobacco (10-18).

Treatment of oral SCC of the tongue usually includes surgery and/or radiation therapy and/or chemotherapy. Few malignancies at present are treated with only one modality. Multidisciplinary care, integrating cancer services (surgery, radiotherapy and chemotherapy) with each other and general services is therefore important (4).

Surgery and radiation therapy have shown favourable results in the treatment of early lesions, with outcomes depending on tumour location and functional changes caused by treatment. Although the cure of early lesions is about 80%, about 10–20% of early lesions, especially those located on the tongue and/or mouth floor, may have subclinical spread to the cervical lymph nodes. These patients may require a combination of surgery and radiotherapy (4). Patients with more advanced tumours, who are not candidates for surgery, are frequently treated with combinations of chemotherapy and radiotherapy. These patients generally have an extremely poor prognosis, because of the inability to fully control these more extensive lesions (4). Observed facto in the service evaluated in this study.

To determine the most effective clinical approach, it is necessary to evaluate factors significantly prognostic of survival. Factors found to have a significant negative effect on patient survival include age >60 years, lesion in the tongue, thickness of the primary tumour between T3 and T4 and clinical TNM stage III or IV (13-17).

Tongue SCC occurs primarily in men aged ≥60 years (10,14-17). Similarly, our study of 412 patients with tongue SCC treated at the Hospital Dr. Luiz Antônio, Natal, Brazil, found that 72.3% of patients with tongue SCC were male, probably because are more exposed to risk factors, including alcohol and tobacco consumption, both of which are more common habits in men than in women (*p*<0,05).

Similar to previous findings (14-17), the patients in our study were of mean age 60.5 ± 14.1 years (range, 19–100 years), with 51.0% aged 0–60 years (50.96%). The highest prevalence by decade was in patients aged 50–59 years (26.21%), in agreement with earlier results (8).

Patient prognosis is improved if oral SCC of the tongue is identified at an early stage. At this stage, however, patients have few or no symptoms, with this absence contributing to delayed diagnosis and treatment (10). For this reason the clinical signs are important for the early diagnosis, so any alteration in the oral cavity that persists for 15 days or more, seek a dental surgeon for evaluation. The self-examination done by the patient may also help in this early diagnosis (3,4).

Treatment is dependent the tumour site, disease stage and general patient health. An earlier diagnosis will require less aggressive treatment, resulting in better function and aesthetic outcomes (8,9). One study reported better overall survival in patients undergoing surgery alone than other forms of therapy (10). In contrast, the 5-year overall survival rate in patients treated with different therapeutic modalities was reaching 24% (10).

According to the Almeida (13) classification, the main factors influencing patient prognosis and survival include the size and location of the primary tumour, regional lymph node involvement, and the presence of distant metastases, with all of these factors included in TNM stage. Prognosis is significantly better in patients with stage I than stage IV tumors (10,18). This study found that tumour size (T stage) and the presence of lymph nodes at diagnosis (N stage) were significant predictors of patient outcomes. This finding indicates that T and N stages can be considered independent factors prognostic of overall survival in this group of patients and that patients without lymph node metastases at initial diagnosis and with T1 or T2 tumours have a higher survival rate (64.92%), confirming previous results (10,18).

After analysing the high mortality rates evidenced in this study and the published reports, it can be concluded that this is a serious disease because of the difficulties of access to adequate treatment, medical professionals often not able to make the appropriate initial diagnosis, and neglect of patients not seeking care but having habits that trigger the disease, corroborating with several surveys (10-18).

Therefore, it is not enough to treat the cancer, but it is also necessary to improve access to health care for the population and raise awareness about the risk factors associated with this disease and related illnesses and train professionals to undertake the diagnosis right. Early detection is the best way to ensure the survival of a cancer patient. It can represent efficacy in the treatment until the patient cures, being one of the main findings of this research.

This study evaluated the clinical and pathological parameters associated with improved survival in patients with oral SCC of the tongue. The absence of lymph node metastases at diagnosis and smaller tumour size were predictive of better survival. Campaigns and projects to encourage and promote the early diagnosis of cancer among health professionals are necessary. In addition, the general public should be aware of the risk factors and prognostic factors for oral SCC of the tongue and of the importance of periodic examinations of the oral cavity.
